# 2024 impact factor increases again and submissions up ~30%

**DOI:** 10.1097/OPX.0000000000002287

**Published:** 2025-08-04

**Authors:** David B Elliott

The leading editorial in this issue is appropriately the summary of the life and career of Jay Enoch by his colleague and fellow giant of optometry academia Gerald Westheimer. However, I could not resist a very short editorial highlighting the improvement in *Optometry & Vision Science’s* 2-year journal impact factor. Clarivate Analytics’ 2-year impact factor is the principal measure by which research journals are evaluated, and journal websites typically state their journal’s impact factor and ranking within their particular field. Despite its limitations,^1^ great importance is placed on positive changes in impact factor and the consequent ranking.^1,2^

*Optometry & Vision Science’s* impact factor has risen from 1.4 in 2022 to 1.6 in 2023 and 1.8 in 2024. With the 3-year plan for the journal including increasing the number of feature issues (supported by a huge increase in the editorial board),^3^ we have already seen a 29.4% increase in submissions in 2024 from 2023 with submissions up again in 2025. Hopefully, we can keep the impact factor rising next year. Thank you to all authors, reviewers, and editors for their support!
